# Autonomous Detection of Humans in Off-Limits Mountain Areas

**DOI:** 10.3390/s24030782

**Published:** 2024-01-25

**Authors:** Jonghoek Kim

**Affiliations:** System Engineering Department, Sejong University, Seoul 05006, Republic of Korea; jonghoek@gmail.com

**Keywords:** autonomous detection of human, off-limits mountains, convolution neural network, motion detection, object classification, object detection, artificial intelligence, time-efficient object detection

## Abstract

This paper is on the autonomous detection of humans in off-limits mountains. In off-limits mountains, a human rarely exists, thus human detection is an extremely rare event. Due to the advances in artificial intelligence, object detection–classification algorithms based on a Convolution Neural Network (CNN) can be used for this application. However, considering off-limits mountains, there should be no person in general. Thus, it is not desirable to run object detection–classification algorithms continuously, since they are computationally heavy. This paper addresses a time-efficient human detector system, based on both motion detection and object classification. The proposed scheme is to run a motion detection algorithm from time to time. In the camera image, we define a feasible human space where a human can appear. Once motion is detected inside the feasible human space, one enables the object classification, only inside the bounding box where motion is detected. Since motion detection inside the feasible human space runs much faster than an object detection–classification method, the proposed approach is suitable for real-time human detection with low computational loads. As far as we know, no paper in the literature used the feasible human space, as in our paper. The outperformance of our human detector system is verified by comparing it with other state-of-the-art object detection–classification algorithms (HOG detector, YOLOv7 and YOLOv7-tiny) under experiments. This paper demonstrates that the accuracy of the proposed human detector system is comparable to other state-of-the-art algorithms, while outperforming in computational speed. Our experiments show that in environments with no humans, the proposed human detector runs 62 times faster than YOLOv7 method, while showing comparable accuracy.

## 1. Introduction

The problem of human detection is to automatically locate people in an image or video sequence, and has been actively researched in the past decade [1,2]. Using an aerial platform, such as an Unmanned Airborne Vehicle (UAV), to perform human detection has been in the attention of researchers for a significant period of time [3].

Our paper is on the autonomous detection of a non-cooperative human in off-limits mountains. Note that we are not interested in the detection of a non-human, such as a wild animal. Our goal is to detect a human in off-limits mountains.

In off-limits mountains, a human rarely exists, thus human detection is an extremely rare event. Suppose one deploys surveillance cameras in armed border areas, in order to detect a non-cooperative human entering the area. Once a non-cooperative human is detected, then the surveillance cameras can let the system operator recognize the human automatically. Considering the maintenance of the surveillance system, it is desirable that the cameras are cheap and their operations are computationally time-efficient.

Due to the advances in artificial intelligence, object detection–classification algorithms based on a Convolution Neural Network (CNN) can be utilized for camera surveillance systems. In object detection–classification algorithms, an object, such as human, is detected and one needs to locate the object in the image. This implies that an object detection and object localization must be performed simultaneously.

There are many papers on object detection–classification algorithms. R-CNN methods [4,5] classified object proposals using deep CNN. R-CNN methods employed several innovations to improve training and testing speed, while also increasing detection accuracy. You Only Look Once (YOLO) [6,7] framed object detection as a regression problem to spatially separated bounding boxes and associated class probabilities. The authors of [8] showed that in an identical testing environment, YOLO-v3 outperforms Single Shot Detection (SSD), and Faster Region based Convolutional Neural Networks (Faster R-CNN), making it the best of the three algorithms. Reference [9] stated that both R-CNN and Fast R-CNN fail to perform real-time detection, but YOLO can perform real-time classification with good speed.

Recently, YOLOv7 and YOLOv7-tiny [10] were developed to improve the detection–classification performance as well as the inference speed of YOLO-based algorithms. The accuracy of YOLOv7 outperforms that of previous YOLOs, such as YOLOv5 or YOLOv6 [11]. However, recently developed object detection–classification algorithms require a Graphics Processing Unit (GPS) for fast processing and are computationally heavy.

Usually, no person enters off-limits mountains, except for personnel guarding the areas. Considering off-limits mountains, there should be no person in general. In off-limits mountains, there can be many wild animals, but detection of wild animals is not the scope of our paper. Therefore, it is not desirable to run object detection–classification algorithms, such as YOLO, continuously, since they are computationally heavy.

This paper addresses a time-efficient human detector system, based on both motion detection and object classification. Here, motion detection is to detect motion by comparing the previous frame with the current one by examining the pixel values. Moreover, object classification is to classify an object inside a given bounding box.

In our paper, we run a motion detection algorithm [12], which is computationally time-efficient. In practice, strong winds can generate the motion of an object. For instance, motion can be detected on various objects, such as tree leaves or squirrels on trees. Since our goal is to detect the motion of a human, the motion of a non-human can be considered as a false alarm for our motion detection algorithm.

In the camera image, we use a feasible human space where a human can appear. For instance, we may have a priori information on humans’ feasible routes. In this case, a feasible human space can be set on humans’ feasible routes. We do not use motion if it is detected outside the feasible human space.

Once motion is detected inside the feasible human space, one enables the object classification, only inside the bounding box where motion is detected. A motion detector in the feasible human space runs much faster than an object detection–classification method, such as YOLO-based methods. Thus, the proposed approach is effective for real-time human detection with low computational loads. As far as we know, no paper in the literature used the feasible human space, as in our paper.

Once motion is detected in the feasible human space, one sets a bounding box on the moving object, followed by classifying the moving object inside the box. For human classification inside the given bounding box, Support Vector Machine (SVM) can be applied to Histograms of Oriented Gradient (HOG) of an object. This method is called HOG detector [13]. However, HOG detector is only suitable for detecting a human with standing upright pose and is sensitive to bounding box size.

In our paper, MobileNetV2 [14] is applied as a CNN model structure for human classification inside the bounding box. Note that MobileNetV2 cannot localize an object in the image. The structure of MobileNetV2 is light, and its operation is fast. MobileNetV2 is selected in our paper, because we focus on time-efficient classification inside the given bounding box. We acknowledge that using MobileNetV2 is not our novel contribution. Applying other recent models, such as MobileNetV3 [15], can further outperform our classification result.

In practice, a human in off-limits mountains may move, while their body is partially occluded. The random erasing method [16] was developed to solve the image occlusion problems. In training the CNN, random erasing approach randomly selects a rectangle region in an image and erases its pixels with random values. In this process, training images with various levels of occlusion are generated, which makes the model robust to occlusion. The random erasing method is simple to implement, since we only need to train various occluded images. Our paper uses the random erasing method for training various occluded human images.

The outperformance of our human detector system is verified by comparing it with other state-of-the-art object detection–classification algorithms (HOG detector [13], YOLOv7 [10], and YOLOv7-tiny [10]) under experiments. This paper demonstrates that our human detector system is much more time-efficient than other state-of-the-art algorithms, while showing comparable accuracy. Our experiments further show that in environments with no humans, the proposed human detector runs 62 times faster than YOLOv7 method, while showing comparable accuracy.

The paper is organized as follows. Section 2 presents the literature review related to this paper. Section 3 describes the proposed human detector system. Section 4 presents experiments of the proposed human detector system. Section 5 presents the conclusions.

## 2. Literature Review

Reference [2] reviewed human detection in surveillance videos and its applications. Object detection could be performed using background subtraction, optical flow and spatio-temporal filtering techniques. Once detected, a moving object could be classified as a human being using shape-based, texture-based or motion-based features. Ref. [2] mentioned that texture-based features, such as HOG detector [13], outperform shape-based, or texture-based methods. Thus, we compare the performance of the proposed human detector with the HOG detector in [13].

In the camera image, we use a feasible human space where a human can appear. We do not use motion if it is detected outside the feasible human space. As far as we know, no paper in the literature used the feasible human space, as in our paper.

For solving the motion detection problem, the optical flow-based object detection technique uses characteristics of flow vectors of moving objects over time to detect moving regions in an image sequence [2,17,18]. Optical flow-based methods can be used to detect independently moving objects even in the presence of camera motion. However, a real-time implementation of optical flow requires a specialized hardware due to the complexity of the algorithm and moderately high frame rate for accurate measurements [2].

For solving the motion detection problem efficiently, various methods have been developed [19,20]. Since one requires cheap surveillance systems with static cameras, it is desirable to use a fast algorithm for motion detection. For motion detection, our paper uses the Gaussian Mixture-based Background Segmentation Algorithm (GMBS) in openCV [21]. We acknowledge that other motion detection algorithm can be used, as long as it runs fast.

For human classification inside a bounding box, Support Vector Machine (SVM) can be applied to a HOG or Scale-Invariant Feature Transform (SIFT) feature of an object [2,13,22,23]. In order to make up for the deficiency of single statistical feature, one can combine more features together for better detection performances [22,23]. However, these detectors are only suitable for detecting a human with standing upright pose and are sensitive to bounding box size.

In our paper, MobileNetV2 [14] is applied as a CNN model structure for human classification inside a bounding box at the feasible human space. In off-limits mountains, a hostile human can crawl, while not standing upright. We argue that CNN structures, such as MobileNetV2 [14] or MobileNetV3 [15], are more extendable than human classifiers in [2,13,22,23], which are specifically designed to classify a pedestrian who usually appears upright in video data.

For instance, for monitoring armed border areas using cameras, CNN structures can be trained to classify a hostile human with weapons, who can be dangerous in armed border area. We can train the CNN network, so that a human with weapons can be distinguished from a human with no weapons. By training CNN structures, we can handle detection of humans, while analyzing the human’s equipment. Human classifiers in [2,13,22,23], which are specifically designed to classify an upright pedestrian, cannot be applied for this equipment detection.

As another example, a lying human can be detected, as long as CNN structures are trained to classify a lying human. One can train CNN structures so that a lying human can be detected. Human classifiers in [2,13,22,23], which are specifically designed to classify an upright pedestrian, cannot be applied to classify a lying human.

The performance of our human detector system is verified by comparing it with other state-of-the-art object detection–classification algorithms (HOG detector [13], YOLOv7 [10], and YOLOv7-tiny [10] ) under experiments. We verify that the proposed human detector system is much more time- efficient than other state-of-the-art algorithms, while showing comparable accuracy.

## 3. Proposed Human Detector System

We address a time-efficient human detector system, based on both motion detection and object classification. At every F>0 frame, one detects a moving object by applying motion detection algorithms. Motion detection algorithms are computationally efficient. Once motion is detected, then one applies the object classification inside the ROI of the moving object.

For motion detection at every *F* frame, one applies the GMBS in openCV [21]. In openCV, the function name is *createBackgroundSubtractorMOG2*. The GMBS calculates the foreground mask performing a subtraction between the current frame and a background model, containing the static part of the scene (everything that can be considered as background given the characteristics of the observed scene).

By setting a sufficiently large *F*, we can further improve the time efficiency of the motion detector. However, setting a too large *F* may disturb the performance of the motion detector, since the static part of the scene may move as time goes on.

Once a moving object is detected, one generates a bounding box surrounding the moving object. The method of obtaining multiple bounding boxes surrounding moving objects is *ConnectedComponentsWithStats* function provided by OpenCV [21]. Due to clutter noise, many small bounding boxes may be falsely generated after ConnectedComponentsWithStats function is applied.

**Figure 1:**
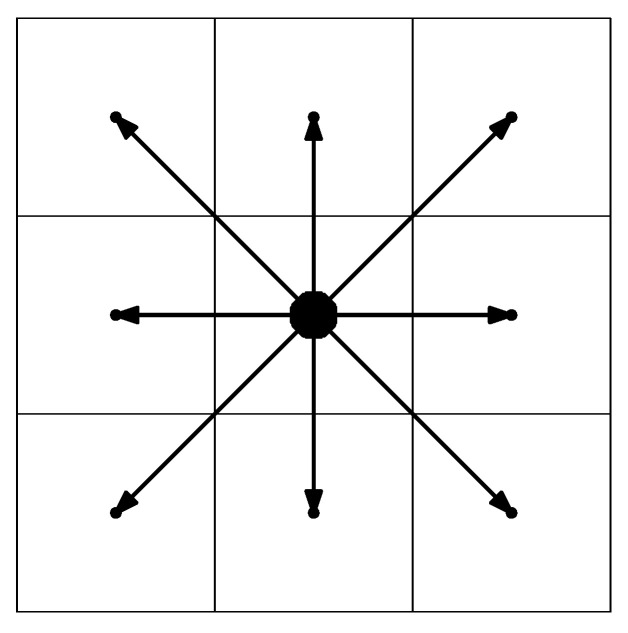
The central cell is connected to all 8 cells surrounding the cell. Here, a connected cell of the central cell is marked with an arrow.

The connectedComponentsWithStats function requires the connectivity option of the function. One sets 8 as the connectivity, which implies that a cell is connected to all eight cells surrounding the cell. In Figure 1, the central cell is connected to all eight cells surrounding the cell. Here, a connected cell of the central cell is marked with an arrow.

The ConnectedComponentsWithStats function returns 2D coordinates of the box center, the width and height of the box, and the pixel area of the box. One then sorts all bounding boxes in the decreasing order of pixel area of each box. Let S denote the set of sorted bounding boxes. Let S[n] denote the *n*-th element (sorted bounding box) in S. S is sorted, such that the pixel area of S[m] is bigger than that of S[n] for any m>n.

**Figure 2:**
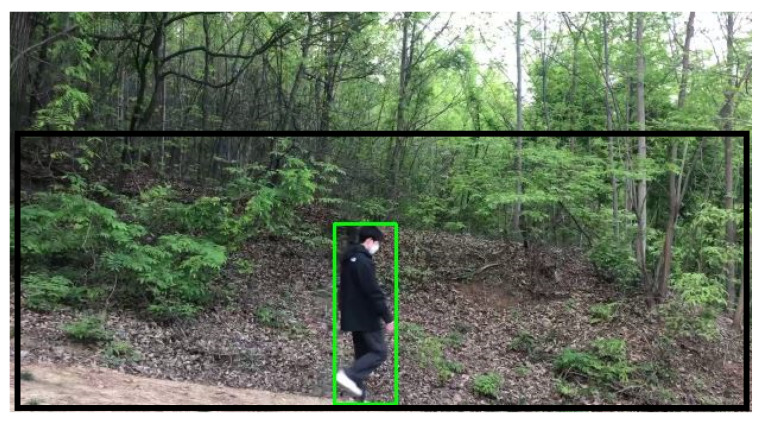
Video file 1. A black rectangle indicates the feasible human space. Once a moving object is detected inside the feasible human space, then one generates the ROI of the moving object. A green rectangle indicates the ROI of the moving object (human) detected using the proposed method.

Once an object’s motion is detected, a bounding box surrounding the object can be detected. In the camera image, we define a *feasible human space*, where a human can appear in the image. We do not use a bounding box if its center is outside the feasible human space. Once a bounding box’s center is detected inside the feasible human space, one enables the object classification, only inside the bounding box. For instance, in Figure 2, a black rectangle indicates the feasible human space.

Let *human bounding box* indicate a box which may contain a human image. Starting from S[1], one checks if there is a bounding box satisfying the following *human bounding box* conditions:

1. The bounding box’s center exists in the feasible human space.
2. The pixel area of the bounding box is smaller than $\delta_{max}$.
3. The pixel width of the bounding box is bigger than $\delta_{min}^{w}$.
4. The pixel height of the bounding box is bigger than $\delta_{min}^{h}$.

Here, $\delta_{max}$, $\delta_{min}^{w}$, and $\delta_{min}^{h}$ are set considering a viable human size in the feasible human space. For instance, we can make a human with various poses move in the feasible human space. Then, we can derive a feasible bounding box size for a human. We say that a human bounding box has a *human* size.

This detection continues until one checks a bounding box which does not have a sufficient number of cells inside it. One uses 300 pixels as this threshold, say Th. This threshold Th is set considering a feasible human size in the feasible human space.

If this threshold Th is not set, then one needs to check every bounding box generated using the connectedComponentsWithStats function. However, this is time-consuming, since there can be a tiny bounding box. Thus, one sets a threshold for detection a human bounding box. See Algorithm 1 for our detection process.
**Algorithm 1** Detect a human bounding box1:Let S denote the set of sorted bounding boxes, returned by the connectedComponentsWithStats function;2:S is sorted, such that the pixel area of S[m] is bigger than that of S[n] for any m>n;3:Let *N* denote the number of all bounding boxes;4:n=1;5:ROI=[];6:**while** the pixel area of S[n] is bigger than Th **do**7:  **if** S[n] is a human bounding box **then**8:  ROI.append(S[n]);9:  **end if**10: *n* = *n* + 1;11:**end while**

**Figure 3:**
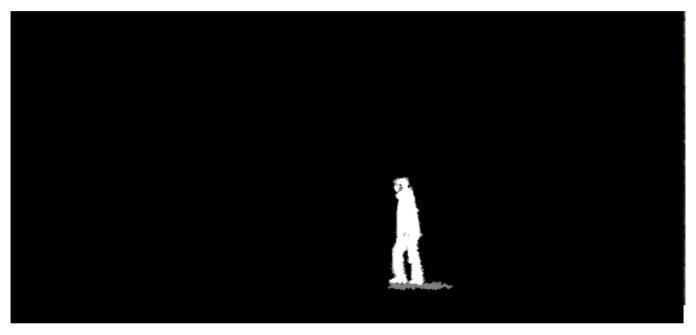
Video file 1. The GMBS and ConnectedComponentsWithStats are applied for detecting a moving object in Figure 2. White area indicates the silhouette of a moving object.

Bounding boxes with human area size are set as *Regions of Interest* (ROIs). Each ROI is set as the object classification input image, so that the object classification is enabled inside the ROI. In Algorithm 1, a bounding box inside the set ROI is used as a ROI for object classification. In Figure 2, a green rectangle indicates the ROI of the moving object (human) detected using the proposed method. In Figure 3, the GMBS and ConnectedComponentsWithStats are applied for detecting a moving object in Figure 2. In Figure 3, white area indicates the silhouette of a moving object. Once a moving object is detected in a frame, then one generates the ROI of the moving object.

### 3.1. Object Classification Inside a ROI

Once a ROI is found, the object classification is enabled inside the ROI. In this paper, MobileNetV2 [14] is applied as a CNN model structure for image classification. The structure of MobileNetV2 is light, and its operation is fast. MobileNetV2 is selected, because we focus on efficient computation.

This paper uses transfer learning of the CNN of MobileNetV2. The CNN’s weighted model, which was pre-trained with COCO dataset [24], is set as the CNN model. For training the network, this paper uses INRIA human dataset [25] with 1240 training images and 597 validation images.

In practice, a human in a border area may move, while their or her body is partially occluded in the woods. The random erasing method [16] was developed to solve the image occlusion problems. In training, random erasing approach randomly selects a rectangle region in an image and erases its pixels with random values. In this process, training images with various levels of occlusion are generated, which makes the model robust to occlusion. Our paper applies the random erasing method [16] to the human dataset, for handling the image occlusion problems.

The batch size was set to 16 as a learning parameter. Since this paper uses the transfer learning, the learning rate was set sufficiently low as $10^{-5}$. Epoch is set to 20, thus the entire learning process repeats 20 times. The learning accuracy of the learning results is 98.67 percent, learning loss is 0.0375, validation accuracy is 98.66 percent, and validation loss is 0.0467. The weight model obtained as a result of learning is stored and used as a binary classifier.

We use a binary classifier, since we only need to classify whether an object is human or not. The classification layer is used to return the probability that the object is human. Let *human probability* denote the probability that the object in the ROI is human.

At each frame, we obtain one or more ROIs. Suppose that there are $N_{k}$ ROIs at frame *k*. Let $R_{n}\left(k\right)$, where $n\in \{1,2,\ldots ,N_{k}\}$, denote the *n*-th ROI at frame *k*. Let $p_{cnn}\left(R_{n}\left(k\right)\right)$ denote the human probability of $R_{n}\left(k\right)$, as the CNN is applied to the ROI $R_{n}\left(k\right)$. In the case where $p_{cnn}\left(R_{n}\left(k\right)\right)>thres$, we assume that $R_{n}\left(k\right)$ contains a human. Here, thres is a tuning parameter, and it can be set as any value in the interval [0,1].

## 4. Test Experiments

For verification of our human detection system, the hardware specifications are as follows: 12th Gen Intel(R) Core(TM) i7-12700K 3.60 GHz, 32 GB. Recall that in the case where $p_{cnn}\left(R_{n}\left(k\right)\right)>thres$, we assume that $R_{n}\left(k\right)$ contains a human. Here, thres is a tuning parameter, and it can be set as any value in the interval [0,1]. In our experiments, we compare between two cases where thres=0.6 and thres=0.3, respectively.

For detecting a human bounding box, we use $\delta_{min}^{w}$ = 10, and $\delta_{min}^{h}=40$, and $\delta_{max}$ = 10,000 pixels in the experiments. This box size can be determined by measuring humans moving in the feasible human space. At every F=5 frame, one detects a moving object by applying motion detection algorithms.

We compare the proposed human detection system with HOG detector [13], YOLOv7 [10], and YOLOv7-tiny [10]. Both YOLOv7 and YOLOv7-tiny have weighted models, which are pre-trained with COCO dataset [10,24]. The training of these algorithms is conducted using the identical dataset, which is used in the proposed algorithm. See Section 3.1.

In our experiments, $Pro_{3}$ indicates the case where our proposed human detection algorithm uses thres=0.3. $Pro_{6}$ indicates the case where our proposed human detection algorithm uses thres=0.6. Both YOLOv7 (3) and YOLOv7-tiny (3) use thres=0.3. Both YOLOv7 (6) and YOLOv7-tiny (6) use thres=0.6. The HOG detector in [13] is only used for human detection.

To analyze the computation time of compared algorithms, we use frame-per-second (FPS). Here, FPS is computed as total number of frames divided by total computation time. In the proposed human detection system, we enable the object classification (MobileNetV2), only inside a bounding box where motion is detected in the feasible object space. The computation time for objection classification (MobileNetV2) is evaluated as FPS−c. Here, FPS−c is computed as total number of objection classification frames divided by total objection classification time. Note that FPS−c is only used in the proposed human detection system.

A classification model performance evaluation index is used to measure the classification accuracy. Among all bounding boxes, True Positive (TP) denotes the number of boxes in which a human is classified as a human. Among all bounding boxes, False Positive (FP) denotes the number of boxes in which a non-human is classified as a human. Among all bounding boxes, False Negative (FN) denotes the number of boxes in which a non-human is classified as a non-human. Among all bounding boxes, True Negative (TN) denotes the number of boxes in which a human is classified as a non-human.

Based on the experiments, the classification accuracy is measured using

(1) $$\begin{array}{c} Accuracy=\frac{TP+FN}{TP+TN+FP+FN}. \end{array}$$

It is desirable that Accuracy in (1) is as close to 1 as possible.

A bounding box can be generated at a non-human, and the box can be falsely classified as a human. This case is associated with FP. In practice, a bounding box can be generated at a partial part of a human, and the box can be falsely classified as a non-human. This case is associated with TN.

As far as we know, there is no open dataset for detecting a person in mountains. For testing our detection system, we made experiment videos, which include a human moving in mountains. As a human moves in mountains, he or she may be occluded due to trees or grass. This makes our experiments challenging, even for the state-of-the-art object detection–classification algorithms. Our test dataset can be provided once requested.

### 4.1. Video File 1 of a Person Moving in Mountains

Our human detection system was tested with a video file, which shows a person passing by in 1920 × 1080 resolution. The video file recorded a person moving in mountains. One human image in the video file is plotted in Figure 2. In Figure 2, a green box indicates the ROI of the moving human detected using the proposed method.

Table 1 shows the classification accuracy and computational load of the proposed human detection system, compared to other state-of-the-art methods. In this table, FPS is used to indicate the computational speed. FPS−c is only applied for the proposed method. Table 1 verifies that the proposed method is comparable to other state-of-the-art YOLO-based methods considering Accuracy. In addition, the proposed method considerably outperforms YOLO-based methods considering FPS (computational speed). See that the accuracy of HOG detector is considerably lower than other methods, since the HOG detector does not use CNN structures.

### 4.2. Video File 2 of a Person Moving in Mountains

**Figure 4:**
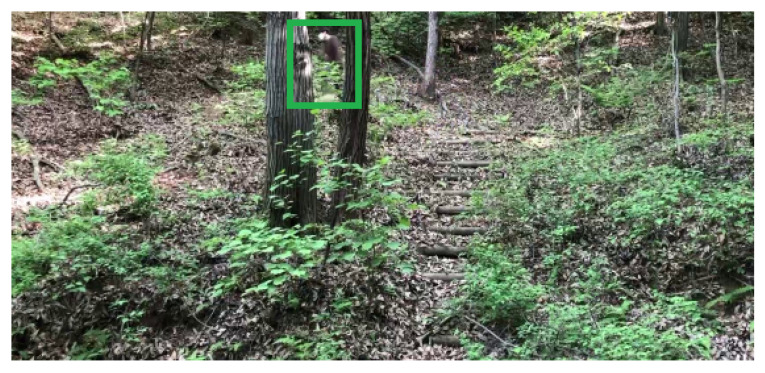
Video file 2. Once a moving object is detected, then one generates the ROI of the moving object. A green box indicates the moving human occluded by trees.

Our human detection system was tested with a video file, which shows a person moving in mountains. One human image in the video file is plotted in Figure 4. Once a moving object is detected, then one generates the ROI of the moving object. In Figure 4, a green box indicates the moving human occluded by trees.

Table 2 shows the classification accuracy and computational load of the proposed human detection system, compared to other methods. Table 2 demonstrates that the proposed method $Pro_{3}$ outperforms other state-of-the-art methods considering Accuracy. Table 2 shows that $Pro_{3}$ performs better than $Pro_{6}$, especially in experiments with occluded human. See Figure 4. The accuracy of HOG detector is considerably lower than other methods, since the HOG detector does not use CNN structures.

### 4.3. Video File 3 of a Person Moving in Mountains

**Figure 5:**
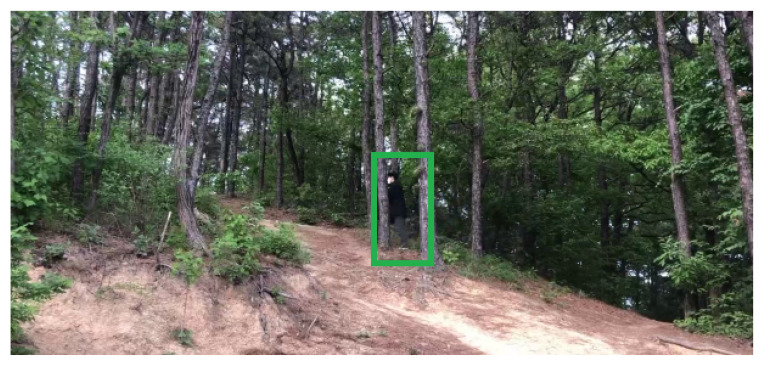
Video file 3. Once a moving object is detected, then one generates the ROI of the moving object. A green box indicates the moving human who is occluded by trees.

Our human detection system was tested with a video file, which shows a person moving in mountains. One human image in the video file is plotted in Figure 5. Once a moving object is detected, then one generates the ROI of the moving object. In Figure 5, a green box indicates the moving human who is occluded by trees.

Table 3 shows the classification accuracy and computational load of the proposed human detection system, compared to other state-of-the-art methods. Table 3 verifies that the proposed method $Pro_{3}$ is comparable to other state-of-the-art YOLO-based methods considering Accuracy. Table 3 shows that $Pro_{3}$ performs better than $Pro_{6}$, especially in experiments with occluded human. See Figure 5. Table 3 further shows that the proposed method outperforms all other methods considering FPS (computational speed). The accuracy of HOG detector is considerably lower than other methods, since the HOG detector does not use CNN structures.

### 4.4. Video File 4 of a Wild Animal in Mountains

**Figure 6:**
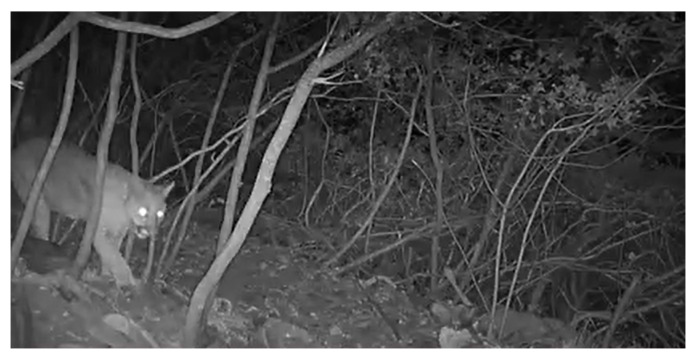
Video file 4 of a wild animal in mountains.

For video surveillance in the off-limit area, IR cameras can be used to detect human activities. IR cameras are useful for detection of humans in dark and cluttered environments. In Video file 4, we consider IR cameras, and we test the case where there is a wild animal in the movie file. See Figure 6.

Considering the case where there is a wild animal, TP and FN are defined as follows. Among all bounding boxes, True Positive (TP) denotes the number of boxes in which a non-human is classified as a non-human. Among all bounding boxes, False Negative (FN) denotes the number of boxes in which a human is classified as a human. Since there is no human in Video file 4, FN = 0.

Table 4 shows the classification accuracy and computational load of the proposed human detection system, compared to other state-of-the-art methods. Table 4 shows that the proposed method $Pro_{6}$ is comparable to YOLOv7(6) considering Accuracy. Table 4 verifies that the proposed method outperforms all other methods considering FPS (processing speed). The HOG detector is not used in Table 4, since the HOG detector cannot be used to detect a non-human.

### 4.5. Video File 5 of No Person in Mountains

In Video file 5, we test the case where there is no human in the movie file. Moreover, there is no moving object in this Video file 5. For this scenario with no human, we use HumanCount as a comparison index. Here, HumanCount indicates the number of cases where a human is detected in the movie file. Considering the case where there is no human in the movie file, it is desirable that HumanCount is as close to 0 as possible.

**Figure 7:**
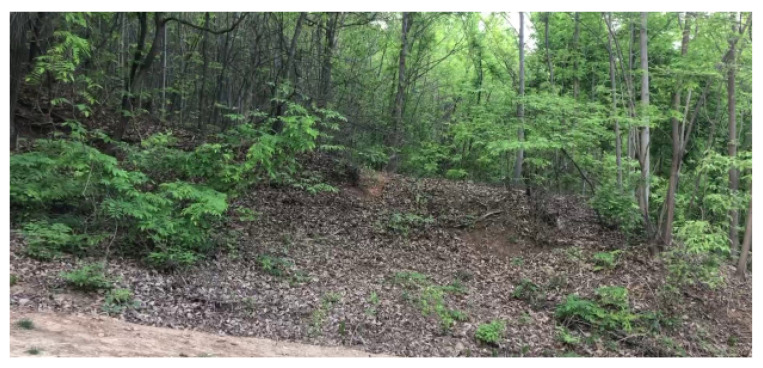
Video file 5 of no person in mountains.

We use a video file of no person in mountains, as plotted in Figure 7. Moreover, there is no moving object in this video file. Table 5 shows the classification accuracy and computational load of the proposed human detection system, compared to other methods. Since there is no human in the video file, HumanCount is zero in the video. Since motion detector runs much faster than an object detection–classification method (e.g., YOLO-based methods), the proposed scheme is suitable for real-time human detection with low computational loads.

Table 5 verifies that our human detector system is much more effective than other state-of-the-art algorithms, especially in the case where there are sparse or no humans. In off-limits mountains, a human rarely exists, hence human detection is extremely rare. Thus, the proposed scheme is preferred in off-limits mountains where there are sparse or no humans.

## 5. Conclusions

Our paper handles the case where the camera system in off-limits mountains detects humans. In off-limits mountains, a human rarely exists, thus human detection is an extremely rare event. We address a time-efficient human detector system, based on both motion detection and object classification.

In the camera image, we use a feasible human space where a human can appear. Our strategy is to enable the object classification, only inside a bounding box where motion is detected in the feasible human space. Since motion detection inside the feasible human space runs much faster than an object detection–classification method, the proposed approach is suitable for real-time human detection with low computational loads. As far as we know, no paper in the literature used the feasible human space, as in our paper.

Experiments showed that the proposed human detector system outperforms other state-of-the-art methods considering both FPS and Accuracy. This paper verifies that the accuracy of the proposed human detector system is comparable to other state-of-the-art algorithms, while outperforming in computational speed.

The proposed detector system can be extended to classify any moving object, such as animals. In the camera image, we can define a *feasible object space* where a moving object can appear. We enable the object classification, only inside a bounding box where motion is detected in the feasible object space. For object classification, we can train the CNN to classify any moving object inside a bounding box. Since motion detection inside the feasible object space runs much faster than an object detection–classification method (e.g., YOLO-based methods), the proposed approach is suitable for real-time object detection with low computational loads.

## Figures and Tables

**Table 1:** Video file 1 comparison (Accuracy and FPS).

Algorithm	Accuracy	FPS (Total Human Frame Number *H*)	FPS−c
$Pro_{3}$	0.99	14 (629)	17
$Pro_{6}$	0.99	16 (629)	17
YOLOv7 (3)	0.94	3 (629)	-
YOLOv7-tiny (3)	0.99	9 (629)	-
YOLOv7 (6)	0.95	1 (629)	-
YOLOv7-tiny (6)	0.99	7 (629)	-
HOG	0.31	19 (629)	-

**Table 2:** Video file 2 comparison (Accuracy and FPS).

Algorithm	Accuracy	FPS (Total Human Frame Number *H*)	FPS−c
$Pro_{3}$	0.93	18 (775)	18
$Pro_{6}$	0.87	16 (775)	18
YOLOv7 (3)	0.73	1 (775)	-
YOLOv7-tiny (3)	0.57	6 (775)	-
YOLOv7 (6)	0.74	1 (775)	-
YOLOv7-tiny (6)	0.8	9 (775)	-
HOG	0	23 (775)	-

**Table 3:** Video file 3 comparison (Accuracy and FPS).

Algorithm	Accuracy	FPS (Total Human Frame Number *H*)	FPS−c
$Pro_{3}$	0.98	33 (339)	19
$Pro_{6}$	0.93	38 (339)	19
YOLOv7 (3)	0.96	1 (339)	-
YOLOv7-tiny (3)	0.94	5 (339)	-
YOLOv7 (6)	0.98	1 (339)	-
YOLOv7-tiny (6)	0.98	10 (339)	-
HOG	0	28 (339)	-

**Table 4:** Video file 4 comparison (Accuracy and FPS).

Algorithm	Accuracy	FPS (Total Frame Number)	FPS−c
$Pro_{3}$	0.87	11 (308)	19
$Pro_{6}$	0.92	13 (308)	19
YOLOv7 (3)	0.64	3 (308)	-
YOLOv7-tiny (3)	0.3	11 (308)	-
YOLOv7 (6)	0.97	3 (308)	-
YOLOv7-tiny (6)	0.67	8 (308)	-
HOG	-	-	-

**Table 5:** Video file 5 comparison (HumanCount and FPS).

Algorithm	HumanCount	FPS (Total Human Frame Number *H*)	FPS−c
$Pro_{3}$	0	62 (0)	15
$Pro_{6}$	0	62 (0)	16
YOLOv7 (3)	0	1 (0)	-
YOLOv7-tiny (3)	0	6 (0)	-
YOLOv7 (6)	0	1 (0)	-
YOLOv7-tiny (6)	0	7 (0)	-
HOG	0	30 (0)	-

## Data Availability

Data is available upon request.
